# *BRCA* mutations in a cohort of Iraqi patients presenting to a tertiary referral center

**DOI:** 10.1186/s12881-019-0885-9

**Published:** 2019-09-05

**Authors:** Chantal Farra, Christelle Dagher, Lama Hamadeh, Nagi El Saghir, Deborah Mukherji

**Affiliations:** 1 Medical Genetics Unit, Pathology Department, American University of Beirut Medical Center, Beirut, Lebanon; 2 Division of Hematology Oncology, Department of Internal Medicine, American University of Beirut Medical Center, Beirut, Lebanon

**Keywords:** Iraq, BRCA1, BRCA2, Breast cancer, Middle East

## Abstract

**Background:**

Unique pathogenic mutations in *BRCA1* and 2 genes have been reported in different populations of patients originating from the Middle East region. Limited data are available for the Iraqi population. For many reasons a large number of Iraqi patients present to Lebanon for medical care. This is the first report of *BRCA* full gene sequencing conducted in a cohort of high-risk patients originating from Iraq.

**Methods:**

This is a retrospective review of Iraqi patients diagnosed with breast or ovarian cancer referred for *BRCA* mutation testing at the American University of Beirut from January 2012 to October 2018.

**Results:**

Of the 42 Iraqi women who underwent genetic testing at our institution, 3 *BRCA* pathogenic variants were found. Two mutations in *BRCA1* c.224_227delAAAG and c.5431C > T and one mutation in *BRCA2* c.5576_5579delTTAA were identified. Three other patients had sequence changes considered as variants of undetermined significance.

**Conclusion:**

In this cohort of high-risk patients, one out of the three pathogenic *BRCA* variants detected has not previously been reported in the Middle Eastern population. Further studies are required to delineate the spectrum of *BRCA* mutations in the Iraqi population.

**Electronic supplementary material:**

The online version of this article (10.1186/s12881-019-0885-9) contains supplementary material, which is available to authorized users.

## Background

The American University of Beirut Medical Center (AUBMC) is a major tertiary referral center in the Middle East. For several reasons including conflict-related deficiencies in healthcare services, a significant number of Iraqi patients seek cancer care at AUBMC [1]. Breast cancer is reportedly the most common primary tumor diagnosed in Iraqi females, with around 3000 cases registered in 2009 [2]. We have recently reviewed the literature regarding *BRCA* mutations reported in the Middle East and North Africa (MENA) region [3], we found a certain number of mutations specific to each country. Very limited information is however available regarding *BRCA* mutations in the Iraqi population.

The aim of our study is to review the data from patients of Iraqi origin referred to our institution for *BRCA* genetic testing. As far as we are aware, this is the first report of whole *BRCA1 and BRCA 2* genes sequencing performed in this population.

## Methods

After Institutional Review Board (IRB) approval, we retrospectively reviewed all cases of Iraqi patients who presented for *BRCA* mutation testing at the Medical Genetics Unit at AUBMC between January 2012 and October 2018. All data were collected from medical charts available at our institution.

QiaAmp DNA Mini kit was used to extract DNA from blood samples. *BRCA1* and *2* genes were amplified using exon-specific primers designed via Primer 3. PCR efficiency was confirmed by amplification on agarose gel 2%. The amplicons were then enzymatically purified using Exonuclease I and Shrimp Alkaline Phosphatase, sequenced and loaded on the Genetic Analyzer (Applied Biosystem ABI 3500). Obtained sequences were analyzed using Seqscape® v2.7 software and compared to the corresponding reference sequences ([4, 5]). ClinVar was used to determine the significance of the variations found [6].

## Results

A total of 42 unrelated Iraqi women were referred for genetic testing performed at AUBMC. The mean age was 47.2 (27–69) years with 10.4 standard deviation. All of the 42 women had a diagnosis of breast cancer, 14 had a positive first-degree family history of breast or ovarian cancer (Table 1). Patients with a diagnosis of breast cancer without a positive first-degree family history were referred for testing for high-risk features in line with National Comprehensive Cancer Network (NCCN) guidelines [7].

**Table 1:** Patients’ demographics

Variable	Number
Number of patients	42
Mean age (years)	47.2 (Range 27–69)
Breast or ovarian cancer family history	14
Breast cancer personal history	42

Two disease causing variants (c.224_227delAAAG and c.5431C > T) in BRCA1 and one (c.5576_5579delTTAA) in BRCA2 was observed in the present study (Table 2) (Additional files 1, 2 and 3). Three variants of unknown significance (VUS) in BRCA1 (c.536A > G, c.1458 T > G, c.1648A > C) were observed in two patients whereas one patient had one VUS in *BRCA2* (c.1075G > A) (Table 3) (Additional files 4, 5, 6, 7, 8, 9 and 10).

**Table 2:** Pathogenic *BRCA* gene mutations detected in the cohort

Gene	Nucleotide Change	AA change	Nomenclature protein	Population previously reported in
*BRCA1*	c.224_227delAAAG	p.Glu75Valfs	343del4	Kurdish Jewish
*BRCA1*	c.5431C > T	p.Gln1811Ter	Q1811X	Greek
*BRCA2*	c.5576_5579delTTAA	p.Ile1859Lysfs	5804del4	Lebanese Chinese

**Table 3:** *BRCA* VUS detected in the cohort

Gene	Nucleotide Change	AA change	Nomenclature protein	Number of patients with mutation
*BRCA1*	c.536A > G	p.Tyr179Cys	Y179C	2
	c.1458 T > G	p.Phe486Leu	F486 L	
	c.1648A > C	p.Asn550His	N550H (benign)	
*BRCA2*	c.1075G > A	p.Glu359Lys	E359K	1

## Discussion

Of the 958 Iraqi patients referred to AUBMC in the past 3 years for treatment of cancer, 14.5% presented for diagnosis, treatment or consultation exclusively for breast cancer [1].

Patients from Iraq receiving cancer care in Lebanon are not covered by third-party insurance resulting in considerable financial constraints related to diagnostic tests and treatment. For this reason, and decreased availability of genetic counseling, we suspect significant underutilization of *BRCA* testing for eligible patients.

We conducted a literature review of previously reported mutations in *BRCA1* and 2 genes detected in patients of Iraqi origin. Very few data have been published on *BRCA* findings in the Iraqi population and of the studies available, none has conducted whole *BRCA1* and 2 genes sequencing.

In 1997, a study investigating the presence of 5 specific *BRCA* mutations in Ashkenazi and non-Ashkenazi Jewish females with breast or ovarian cancer identified the presence of the Ashkenazi Jewish common mutation *BRCA1* 185delAG, for the first time in an Iraqi patient with no known Ashkenazi ancestry [8] The *BRCA1* 185delAG has been identified in several other ethnic groups [9]. One other study used PCR amplification with 3 specific primer pairs to investigate the prevalence of 3 specific mutations (*BRCA1* 185delAG, *BRCA1* 5382insC and *BRCA2* 6174delT) in a cohort of Patients in the Hilla region of Iraq however these results have not been validated [10].

In the present study 3 *BRCA* pathogenic variants were detected by whole gene sequencing performed for clinical reasons. The *BRCA2* c.5576_5579delTTAA, which was previously identified in one patient of Lebanese origin [11], is also reportedly a founder mutation in the Chinese population [12]. The *BRCA1* c.224_227delAAAG found in our study was previously identified in in 2 out of 53 patients with breast cancer of Kurdish Jewish descent [13].

The third pathogenic variant found in our cohort, *BRCA1* c.5431C > T has been documented in 3 unrelated patients with breast cancer of Greek origin [14]. It has not to date been reported in the Middle East region.

## Conclusion

In a cohort of high-risk patients for hereditary breast or ovarian cancer of Iraqi origins referred for *BRCA* testing at our institution, one out of three identified pathogenic variants has not previously been reported in the Middle East region. Because of the heterogeneity of the variants reported to date in the different populations that have been studied in the region, a unified and affordable, panel that would target specific variants cannot be recommended at this stage. Further studies utilizing whole-gene sequencing approaches are required to formulate evidence-based guidelines for *BRCA* and other cancer related genetic analyses in the region.

## Additional files

Additional file 1: *BRCA1* c.224_227delAAAG- 16-018 BC (JPG 170 kb)

Additional file 2: *BRCA1* c.5431C > T-16-053 BC (JPG 238 kb)

Additional file 3: *BRCA2* c.5576_5579delTTAA-13-046 BC (JPG 178 kb)

Additional file 4: *BRCA1* VUS c.536A > G- 15-050 BC (JPG 179 kb)

Additional file 5: *BRCA1* VUS c.536A > G- 14-072 BC (JPG 175 kb)

Additional file 6: *BRCA1* VUS c.1458 T > G- 14-072 BC (JPG 244 kb)

Additional file 7: *BRCA1* VUS c.1458 T > G- 15-050 BC (JPG 223 kb)

Additional file 8: *BRCA1* VUS c.1648A > C-14-072 BC (JPG 223 kb)

Additional file 9: *BRCA1* VUS c.1648A > C- 15-050 BC (JPG 220 kb)

Additional file 10: *BRCA2* VUS c.1075G > A- 17-059 BC (JPG 244 kb)

## Data Availability

The datasets used and analyzed during the current study are available from the corresponding author on reasonable request.

## Abbreviations

AUBMC: American University of Beirut Medical Center
IRB: Institutional Review Board
MENA: Middle East and North Africa
