# Supplementary figures and images for: BRCA mutations in a cohort of Iraqi patients presenting to a tertiary referral center
Source: BMC Med Genet. 2019 Sep 5;20:154. doi: 10.1186/s12881-019-0885-9 (PMC6728935; doi:10.1186/s12881-019-0885-9)

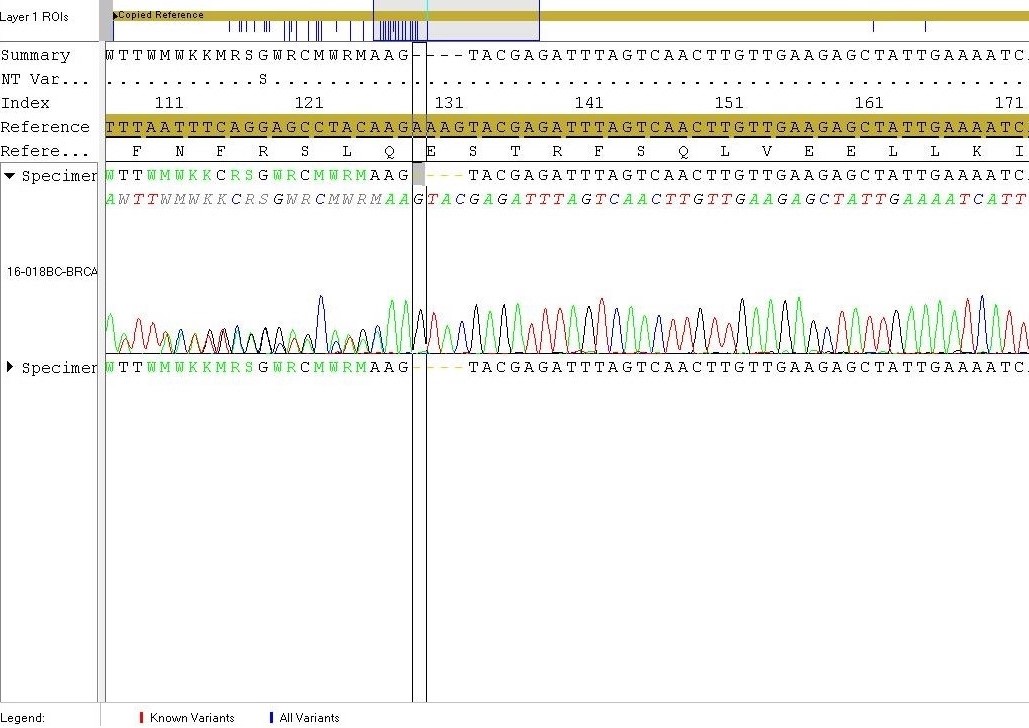

Supplement: Supplementary file 1 — BRCA1 c.224_227delAAAG- 16-018 BC (JPG 170 kb) [file 12881_2019_885_MOESM1_ESM.jpg]

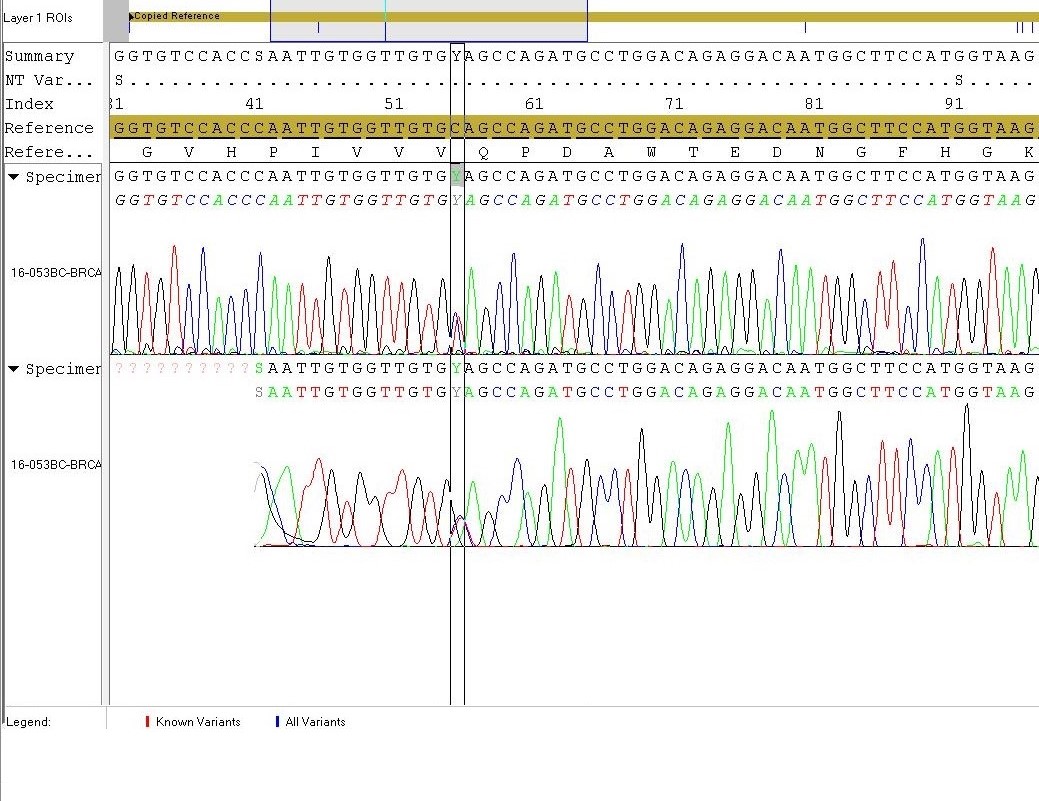

Supplement: Supplementary file 2 — BRCA1 c.5431C > T-16-053 BC (JPG 238 kb) [file 12881_2019_885_MOESM2_ESM.jpg]

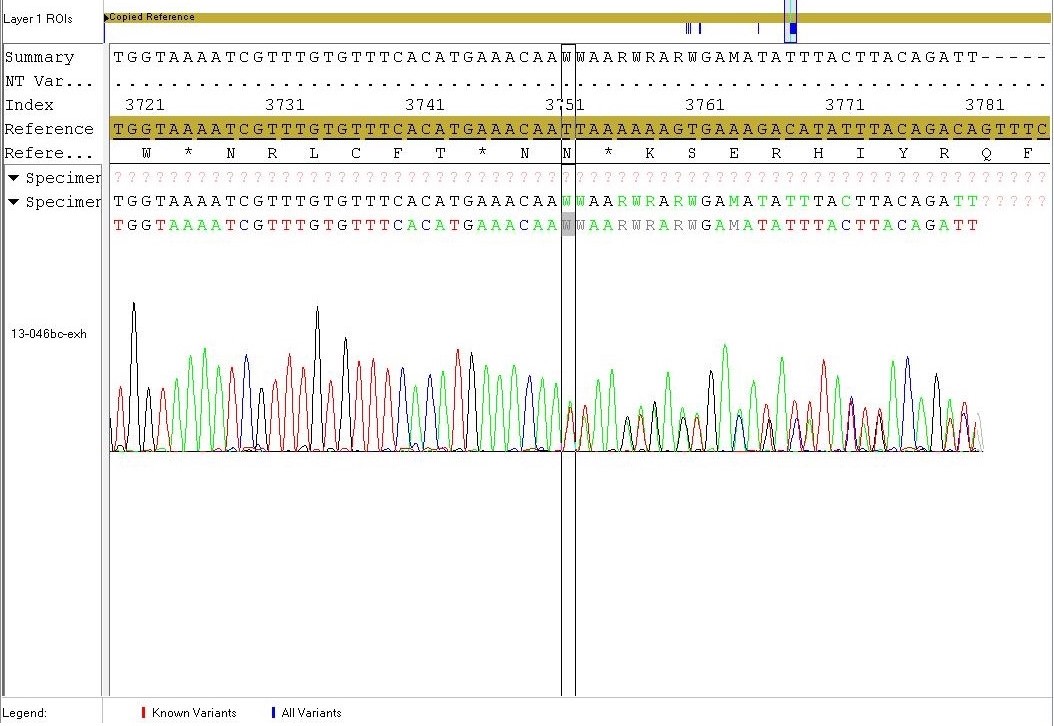

Supplement: Supplementary file 3 — BRCA2 c.5576_5579delTTAA-13-046 BC (JPG 178 kb) [file 12881_2019_885_MOESM3_ESM.jpg]

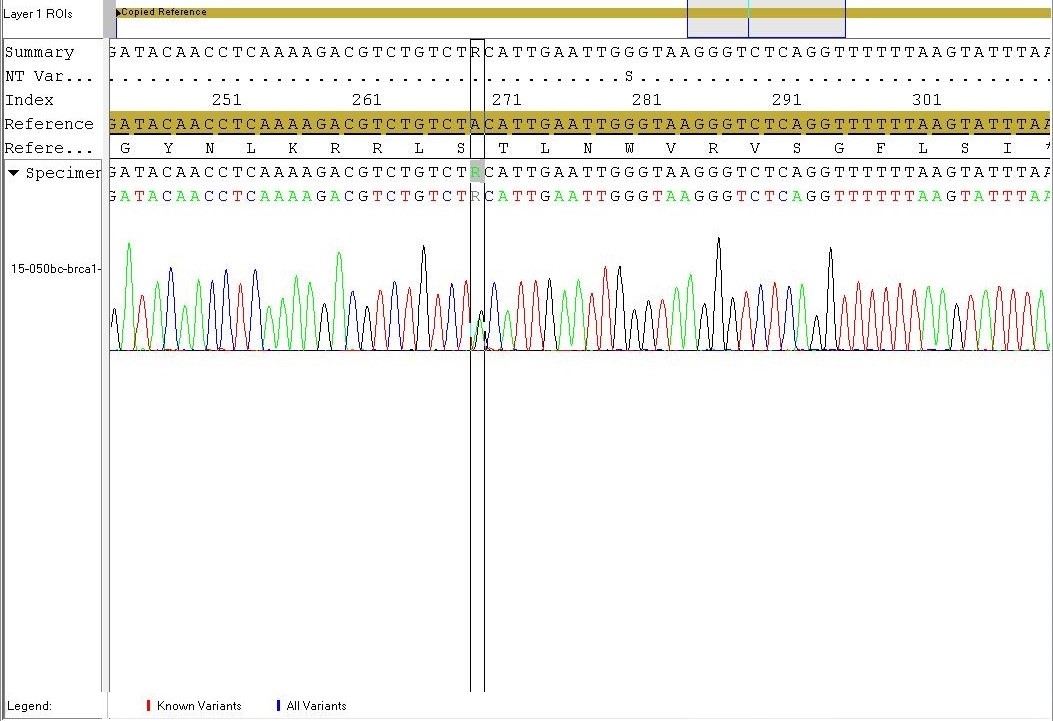

Supplement: Supplementary file 4 — BRCA1 VUS c.536A > G- 15-050 BC (JPG 179 kb) [file 12881_2019_885_MOESM4_ESM.jpg]

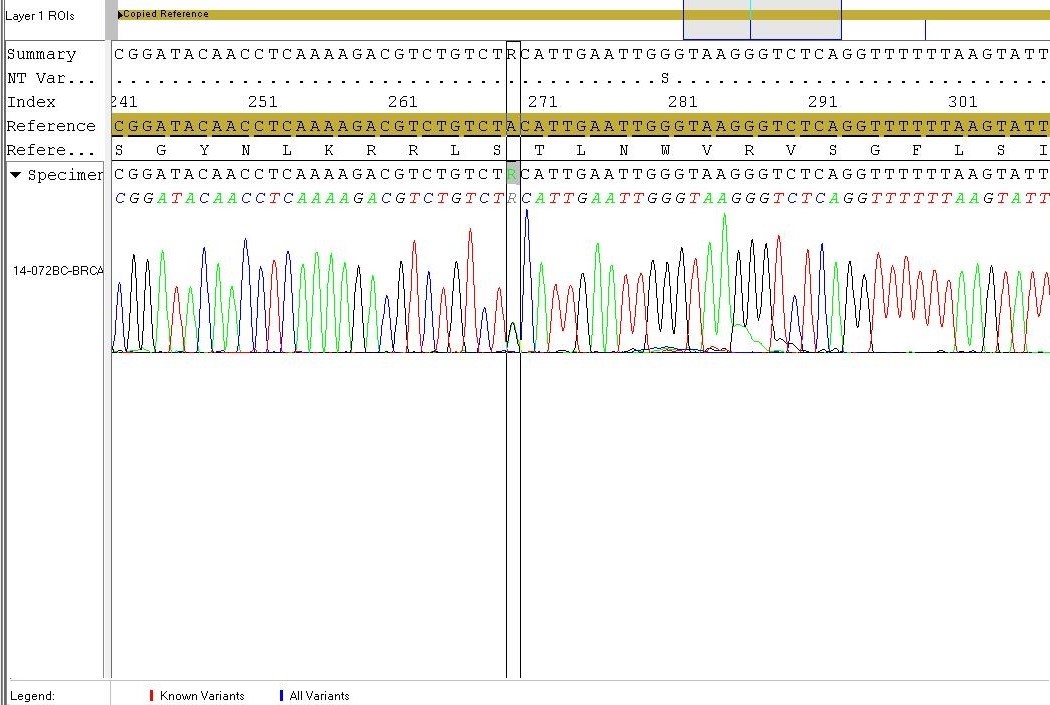

Supplement: Supplementary file 5 — BRCA1 VUS c.536A > G- 14-072 BC (JPG 175 kb) [file 12881_2019_885_MOESM5_ESM.jpg]

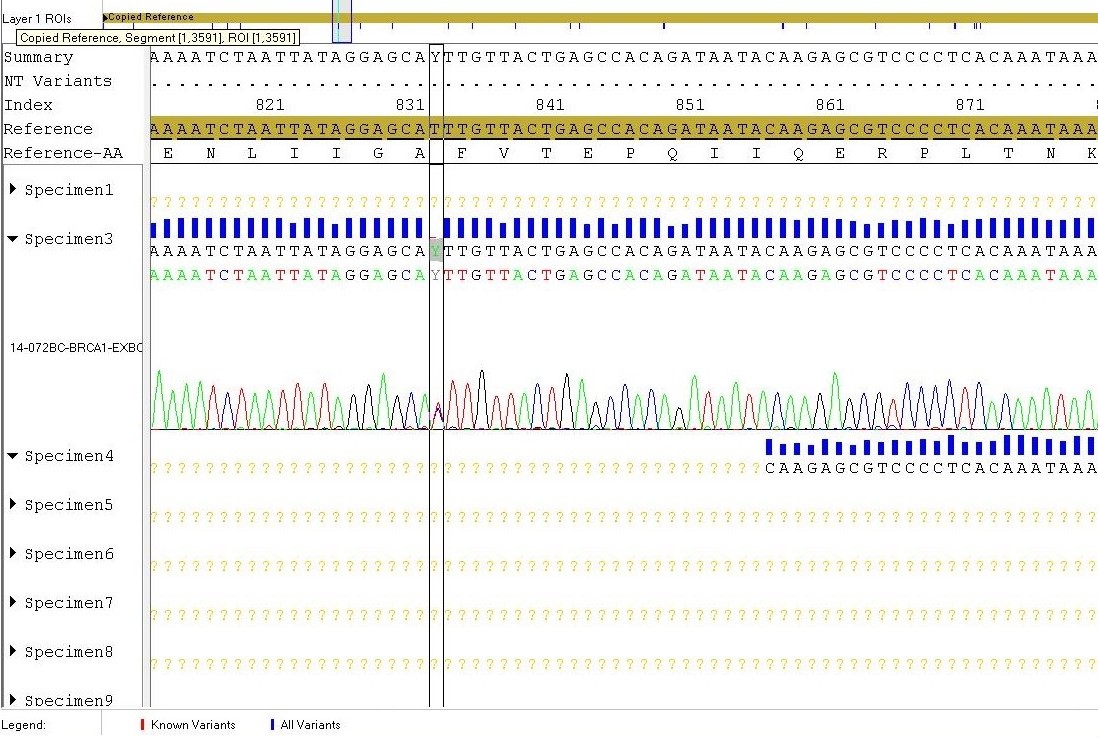

Supplement: Supplementary file 6 — BRCA1 VUS c.1458 T > G- 14-072 BC (JPG 244 kb) [file 12881_2019_885_MOESM6_ESM.jpg]

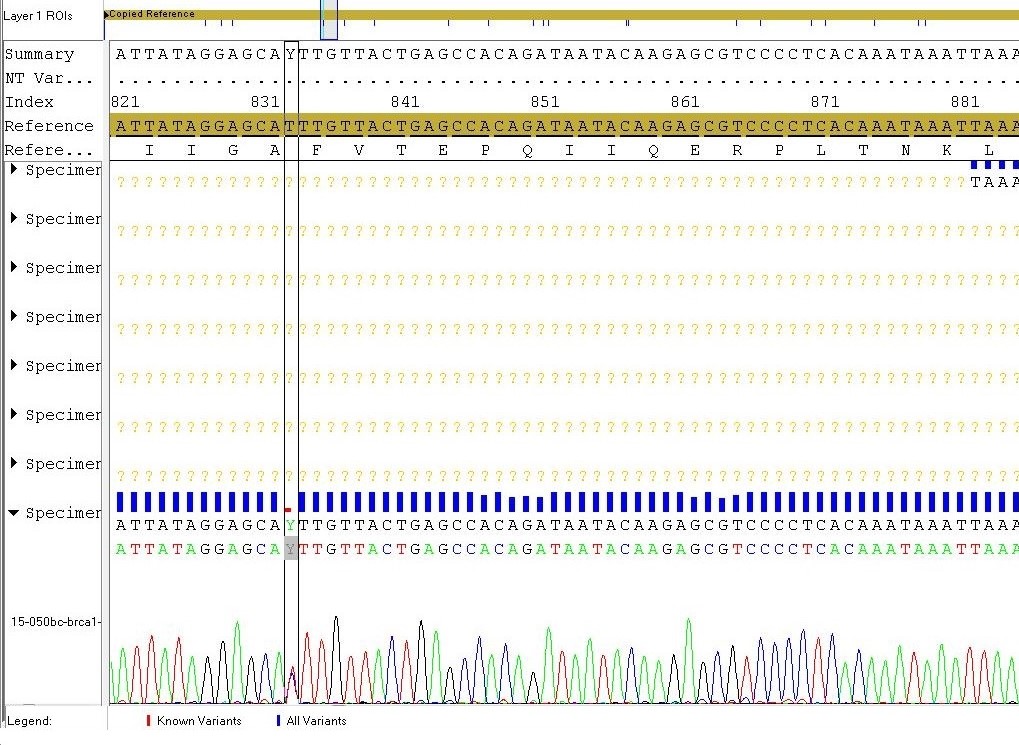

Supplement: Supplementary file 7 — BRCA1 VUS c.1458 T > G- 15-050 BC (JPG 223 kb) [file 12881_2019_885_MOESM7_ESM.jpg]

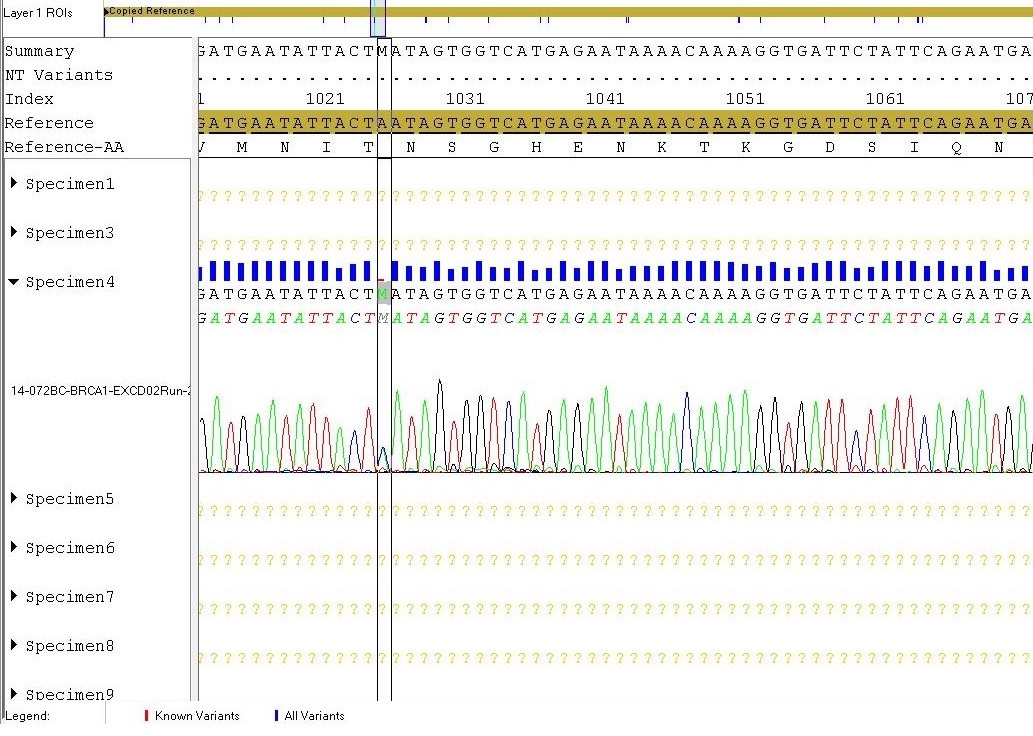

Supplement: Supplementary file 8 — BRCA1 VUS c.1648A > C-14-072 BC (JPG 223 kb) [file 12881_2019_885_MOESM8_ESM.jpg]

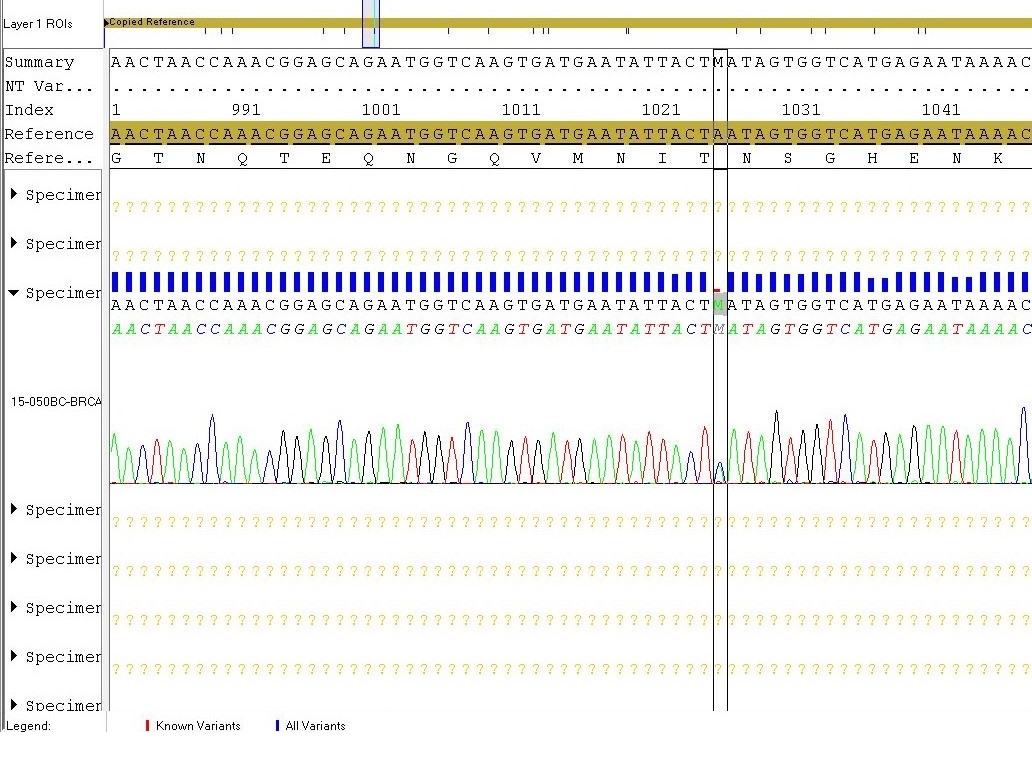

Supplement: Supplementary file 9 — BRCA1 VUS c.1648A > C- 15-050 BC (JPG 220 kb) [file 12881_2019_885_MOESM9_ESM.jpg]

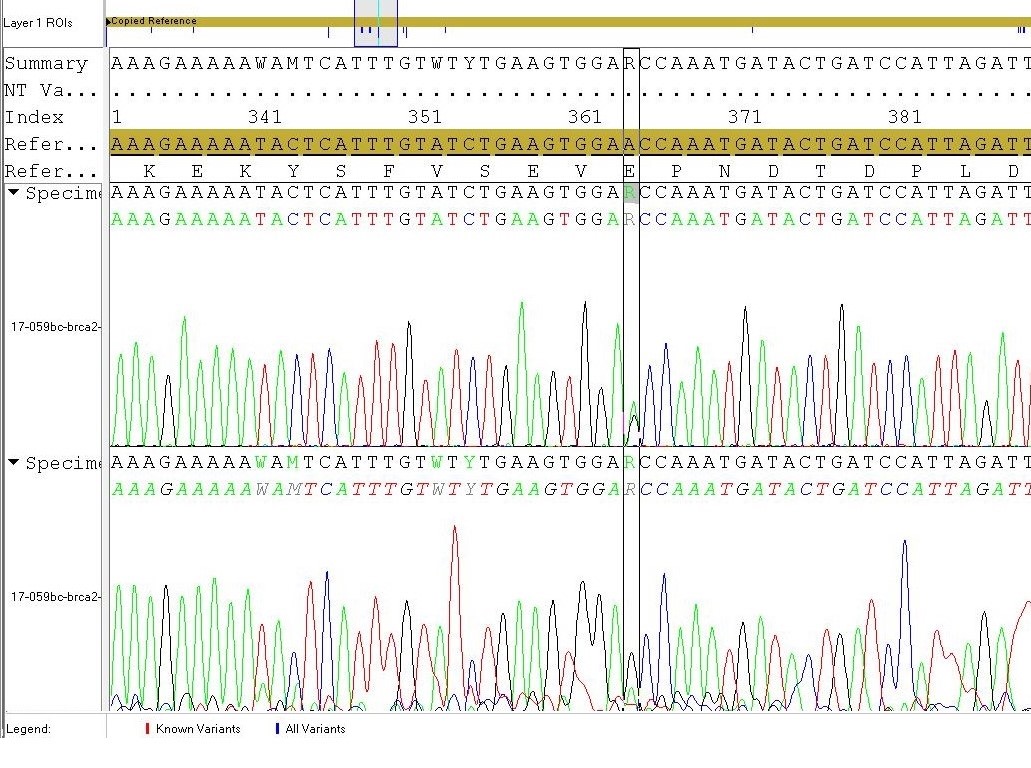

Supplement: Supplementary file 10 — BRCA2 VUS c.1075G > A- 17-059 BC (JPG 244 kb) [file 12881_2019_885_MOESM10_ESM.jpg]
